# Cogmed Working Memory Training Presents Unique Implementation Challenges in Adults With ADHD

**DOI:** 10.3389/fpsyt.2018.00388

**Published:** 2018-08-28

**Authors:** Enitan T. Marcelle, Erica J. Ho, Michelle S. Kaplan, Lenard A. Adler, F. Xavier Castellanos, Michael P. Milham

**Affiliations:** ^1^Department of Psychology, University of California, Berkeley, Berkeley, CA, United States; ^2^Center for the Developing Brain, Child Mind Institute, New York, NY, United States; ^3^Nathan Kline Institute for Psychiatric Research, Orangeburg, NY, United States; ^4^Department of Psychology, Yale University, New Haven, CT, United States; ^5^ADHD and Behavior Disorders Center, Child Mind Institute, New York, NY, United States; ^6^Department of Psychiatry, Langone Medical Center, School of Medicine, New York University New York, NY, United States; ^7^Department of Child and Adolescent Psychiatry, Hassenfeld Children's Hospital at NYU Langone, New York, NY, United States

**Keywords:** ADHD, working memory, working memory training, adults, Cogmed, non-psychopharmacological treatment

## Abstract

Cogmed Working Memory Training (CWMT), an online cognitive training program developed for children, is an increasingly popular non-pharmacological intervention for ADHD amongst all ages, despite limited supporting evidence. The initial objective of the present work was to examine the short- and long-term impacts of CWMT on brain function in adults with ADHD. However, during the conduct of our study, we experienced multiple levels of failures in recruitment and retention that signaled potential concerns about the suitability of CWMT for adults with ADHD. This perspective piece aims to describe the difficulties we encountered in the context of studies examining the efficacy of CWMT in comparable populations. We trace these difficulties to the limited tolerability of the current CWMT structure for adults with ADHD, and review similar limitations in the literature. We suggest that efficacy of CWMT in children may be due in large part to close monitoring and scaffolding provided by clinicians and caregivers. For CWMT to have viability for widespread use in adults, greater support and structure will be needed for users to improve the likelihood of adherence. We discuss implications and considerations for future efforts in both research and clinical practice.

## Introduction

Attention-deficit/hyperactivity disorder (ADHD) is one of the most common neurodevelopmental disorders in children, characterized by impairing levels of inattention and/or hyperactivity-impulsivity. Longitudinal studies have found that impairing ADHD symptoms persist into adulthood in up to 65% of cases (1, 2). Across ages, individuals with ADHD experience difficulties in occupational, social, and academic functioning (3–5). In adulthood, individuals with ADHD exhibit higher rates of substance abuse, motor vehicle accidents, accidental injury to self, non-suicidal self-injury, and suicide (6). In short, ADHD is increasingly recognized as entailing poor functioning across the lifespan.

When treating adults with ADHD, clinicians commonly encounter additional obstacles relating to adherence and efficacy. Stimulants, which are among the most effective treatments for ADHD in children, have been suggested to be less efficacious in adults (7, 8), with 20–50% of adults with ADHD not responding to medication (9). Among responders, the efficacy of psychopharmacological interventions is reduced by poor medication adherence (10) resulting from difficulties with self-management (e.g., organization, maintenance of schedule) characteristic of this clinical population (11, 12). Psychosocial therapies, another potential treatment for ADHD (7), are limited by scarcity of accessible treatment providers, leaving the majority of adults diagnosed with ADHD without effective care. As a result, there is a growing demand for non-pharmacological interventions for adults with ADHD (13) that are efficacious and can easily be scaled.

Cognitive training has gained increasing attention for the potential to overcome these obstacles, leveraging computerized platforms to decrease challenges of delivery. With regard to ADHD, working memory (WM) has emerged as a particularly attractive target for computerized training due to its demonstrated impairment in ADHD (14–16). WM is defined as the ability to both store and manipulate transient mental information (17). Once thought to be static, investigation into the neuroplasticity of WM has revealed that its capacity and efficiency can be expanded through targeted cognitive training (18, 19). As such, interest in the clinical utility of self-paced, home-based WM training programs has burgeoned in recent years.

Of the many WM training programs that exist, Cogmed Working Memory Training (CWMT) has become the most widely empirically researched intervention (20, 21). The CWMT program was initially developed by a team of neuroscientists aiming to investigate the neuroplasticity of WM. Carried out in 25 computerized sessions (each lasting 30–45 min) over the course of 5 weeks, CWMT uses an adaptive training model to update task difficulty in response to performance. This model ensures the user is consistently stretching the limits of their WM capacity, providing benefits above and beyond competing “one size fits all” training approaches. Cogmed users are instructed to complete five sessions a week over the course of 5 weeks. Although Cogmed users are encouraged to complete these trainings at the same time each day, users are able to complete trainings at any time. Each session consists of eight exercises that target various aspects of working memory. Cogmed advertises that users can complete sessions at home, work, school, or anywhere internet access is available. While Cogmed users complete the individual training sessions on their own, each user is paired with a trained “Cogmed Qualified Coach” who has completed all training per Cogmed requirements. This coach is a mental health professional whose goal is to monitor users' progress through the program, as well as provide support, structure, and motivation. CWMT is marketed as a uniquely positioned therapeutic modality by Pearson, Inc., which claims that “Cogmed is based on strong scientific research” and that “over 100 peer-reviewed studies support the efficacy of Cogmed with children and adults across a variety of applications” (22). Despite the enthusiasm, concerns about the efficacy of this approach have been raised (20, 23). Significant questions regarding efficacy, far transfer effects, long-term impact on neural functioning, and appropriateness of common probes of near transfer effects exist (24). Furthermore, claims regarding the utility of CWMT in adults have a limited basis, as CWMT was developed for and primarily tested in children. A relatively small portion of the extant literature has examined the efficacy of Cogmed in older populations, with just three research groups focusing on adults diagnosed with ADHD (24–27).

The goal of the present perspective piece is to draw attention to questions regarding the feasibility of using CWMT in the treatment of adults with ADHD. These concerns emerged from a failed study of CWMT, which aimed to examine the short- and long-term impact of working memory training on brain function in adults with ADHD, using functional magnetic resonance imaging (fMRI). We focus on the acceptability and practicality of the intervention, defined by Bowen et al. (28) as the extent to which a program is deemed suitable, satisfying, or attractive to participants, and the ability of participants to carry out program activities. In the conduct of our research we experienced failures in both participant recruitment and retention, drawing attention to concerns about the acceptability and practicality, as measured by rates of enrollment and completion of CWMT for adults with ADHD. Below, we first provide a description of the study design. We then discuss specific challenges that arose in the conduct of the study and place our experience in the context of the larger literature.

## Planned study design

### Participants

Participants were recruited through local newspapers, internet-based advertising, college and university learning centers, and ADHD support groups. Participants taking psychotropic medications were included provided that their medication dosage had been stable for 1 month prior to study enrollment, and remained stable throughout their participation. Inclusion criteria were as follows: (1) male or female between 18 and 40 years of age; (2) capacity to provide informed consent; (3) right-handed; (4) fluent in English; and (5) met criteria for ADHD (current and in childhood), either inattentive or hyperactive/impulsive type, as established through the Adult ADHD Clinical Diagnostic Scale (ACDS) v 1.2 (29). Exclusion criteria were as follows: (1) history of severe head trauma; (2) lifetime history of pervasive developmental, bipolar, psychotic, or substance disorders; (3) current depressive disorder; (4) Full scale *IQ* < 85, as established through the Wechsler Abbreviated Scale of Intelligence (WASI); (5) contraindications to MRI; and (6) a change in psychotropic medication during study participation. All research procedures were approved by the Institutional Review Board at the Nathan Kline Institute.

**Figure 1:**
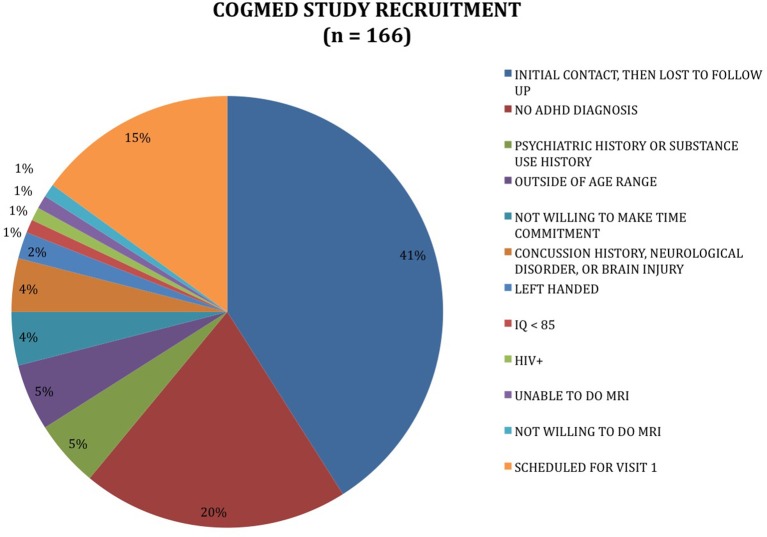
Recruitment outcomes for planned study.

Outreach generated a total of 166 contacts; 13 individuals met full criteria for participation and were provided CWMT accounts (see Figure 1).

### Procedures

The study consisted of four separate visits. In the first, we obtained demographic information and medical history, administered a semi-structured diagnostic interview (Structured Clinical Interview for DSM-IV (SCID); Adult ADHD Self-Report Scale (ASRS) v1.1; Behavior Rating Inventory of Executive Function (BRIEF)-Adult Version), abbreviated psychological (Wechsler Abbreviated Scale of Intelligence, second edition; Wechsler Individual Achievement Test (WIAT)-abbreviated, second edition) and neurocognitive testing (NIH EXAMINER Battery; n-back and temporal discounting tasks). Socioeconomic status (SES) was measured using the Hollingshead Four-Factor Index of Socioeconomic Status. The second visit took place within 2 weeks before beginning CWMT and consisted of an MRI scan. The third and fourth visits took place 1 week after and 1 month after completing CWMT, respectively, and each consisted of an MRI scan as well as abbreviated psychological and neurocognitive testing. Each participant was provided with a trained CWMT coach, a Licensed Master Social Worker, who gave verbal and written feedback and monitored progress through the program on a weekly basis. Coaching content included reviewing daily and weekly schedules to choose consistent daily training times, building in rewards around training, and offering praise when positive patterns in training occurred. Participants unable to complete at least 20 computerized training sessions over a 5-week period were excluded from the study due to dosage-dependent nature of CWMT effects.

Participants were compensated for in-person assessments (initial visit: $75; MRI sessions: $50; follow-up visits: $25 each). All aspects of CWMT, including coaching, were provided at no cost to participants.

## What went wrong

**Figure 2:**
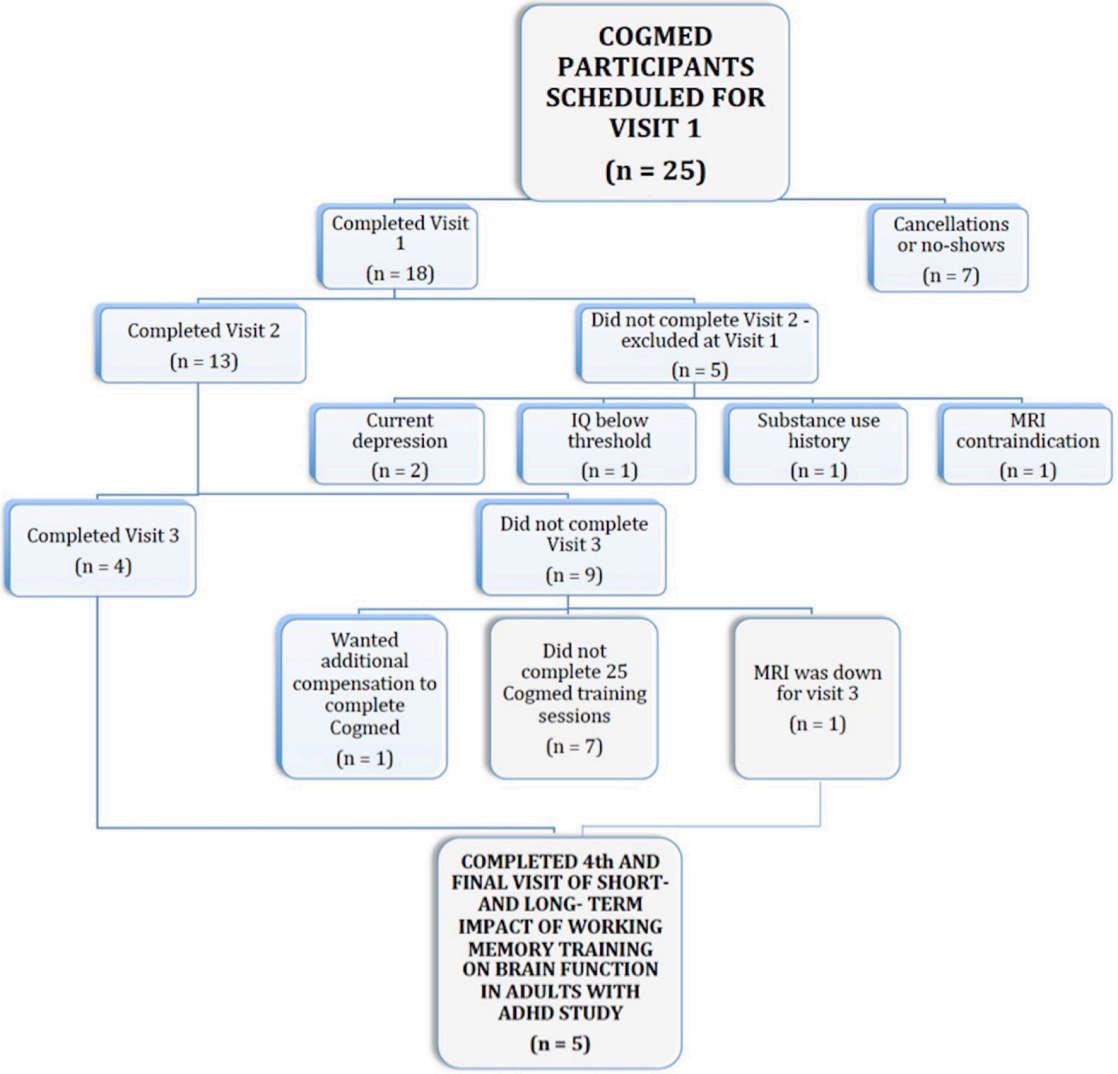
Flowchart delineating reasons for participant attrition.

Our high attrition rate and final sample size prevented us from carrying out our intended aims. Of the 13 participants who began CWMT, only 38% (*n* = 5) completed the program (see Figure 2), falling short of the suggested 10 participants needed for acceptable statistical power in pre- vs. post-intervention fMRI designs (30, 31). Our trained phone coach experienced significant difficulty motivating participants to remain engaged and to complete CWMT. Issues connecting with participants for phone coaching resulted in many coaching sessions being conducted via email. Furthermore, despite being offered clinic space or library space as needed, several of our participants also reported that securing daily access to the internet was a major obstacle. Finally, participants expressed displeasure with training length and intensity. Qualitative exit data collected revealed that CWMT took much longer than half an hour for participants to work through and that the training was perceived as excessively taxing. One participant provided the following rationale for discontinuing: “It is really hard to put aside an hour and a half for Cogmed, and the training has been very frustrating to complete.” Indeed, although CWMT is marketed as requiring 30–45 min per day, our participants took an average of 55 min (*SD* = 19.2) to complete each session. We hypothesize that the prolonged duration of each session resulted in part from the executive functioning impairments that are characteristic of ADHD.

## Intolerability of CWMT in adults with ADHD

Given that our initial sample consisted of only 13 participants, it is possible that unappreciated biases in our sample selection may somehow explain the 62% drop-out rate we observed. *Post-hoc* (*t*-tests and chi-squared tests) analysis did not reveal any differences between completers and non-completers in age, sex, race, executive function capabilities, SES, IQ, symptom severity, medication status, or pre-training self-reported motivation (*p-*values ranging from 0.08 for sex to 0.98 for motivation, see Table 1); however, these findings are limited with respect to statistical power. Of note, participants who dropped out all completed at least three sessions (and an average of 10; *SD* = 4.4), indicating that the decision to discontinue was not precipitous in most cases. As such, it appears that CWMT did not meet participants' desired levels of acceptability or practicality, and that participants experienced CWMT as more time consuming than advertised. Due to difficulties intrinsic to ADHD, this population found multiple aspects of CWMT extraordinarily challenging. For example, one diagnostic criteria for ADHD, endorsed by 100% of our participants, is the tendency to avoid and dislike sustained mental effort. Additionally, the requirement for sufficient self-organization to perform massed at-home training bears on another core disability in ADHD.

**Table 1:** Demographic characteristics of study participants by completion status.

	**Cogmed completion status**		
	**Completers**	**Non-completers**	
	**(*n = 5*)**	**(*n = 8*)**	***p***
Age (years)	26.6 (6.3)	28.9 (4.9)	0.476^b^
Race, *n* (%)			0.460^c^
White	3 (60)	4 (50)	
Black	1 (20)	3 (37.5)	
Asian	1 (20)	0 (0)	
Other	0 (0)	1 (12.5)	
Sex, *n*(%)			0.086^c^
Male	1 (20)	6 (75)	
Female	4 (80)	2 (25)	
SES	33.8 (18.5)	46.3 (15.5)	0.215^b^
WASI	109.2 (13.5)	110.5 (11.5)	0.856^b^
BRIEF	63.2 (15.3)	67.2 (9.4)	0.646^b^
ASRS	39.6 (14.4)	36.4 (13.5)	0.691^b^
Medication Status			0.510^c^
Medicated	2 (40)	2 (25)	
Unmedicated	3 (60)	6 (75)	
Motivation	37.4 (6.3)	37.3 (9.2)	0.989^b^

a *Values are expressed as mean(SD), unless otherwise specified*.

b *2-sample t-test*.

c *Chi-squared test*.

To validate our concerns about CWMT, we turned to the literature. While no systematic review examining CWMT attrition in comparable populations exists, we did find a number of studies that provided insights. In particular, three research groups (25, 26, 32) have specifically examined the suitability and efficacy of CWMT for adults with ADHD. Mawjee and colleagues aimed to compare standard-length (45 min-the duration used in our study) and shortened-length (15 min) CWMT. Standard and shortened trainings were similar in all regards except session length. Mawjee et al. focused on college aged students recruited through student disability services. Weekly 30-min phone sessions with a certified CWMT coach were provided. Both studies led by Mawjee et al. studies reported high attrition rates overall, with higher drop-out rates for the standard-length group relative to the shortened-length and waitlist control groups [22, 6, and 9%, respectively, (26); 56, 25 and 25% 33]. Mawjee et al. (26) mentions compensating participants for study completion whereas the later study Mawjee et al. (33) does not; this may have contributed to variability in rates of retention. This pattern of attrition, however, provides evidence in support of possible concerns about session duration. Of note, Mawjee et al. used Cogmed RM, which resembles a video game and is designed for children aged 7 and up, and not the adult Cogmed QM platform, which is arguably less engaging. In both studies, the shortened- and standard-length versions of Cogmed conferred comparable WM improvement, leading Mawjee and colleagues to conclude that shortened-length CMWT yields higher rates of adherence. These findings also raise the enticing possibility of using Cogmed RM in adults as a ready-made way of increasing the appeal and feasibility of the training experience.

Two recent studies provide somewhat more optimistic estimates of tolerability. First, Dentz et al. (25) investigated the efficacy of CWMT in ADHD-diagnosed adults recruited through a mental health clinic. However, the earlier study Cogmed coaching in this study was performed by a research assistant rather than a clinician. Of 55 participants, only 11 (20%) chose to withdraw from the study, citing difficulties with time constraints and organization. Similar to our experience, Dentz et al., also found no differences between completers and withdrawers on measures of age, education, sex, SES, and other dependent variables. Second, Gropper et al. (32) sought to investigate feasibility of Cogmed within a population of college students with ADHD or learning disabilities. This study did *not* see the high attrition rates that our present study and Mawjee et al. experienced, with 87% of students completing training. In attempting to put the pieces together among these studies, it is not clear what factor(s) may be driving differences in drop-out rates across studies. Differences in age-group, recruitment setting, and qualifications of the CWMT coach provided do not cleanly explain completion rate disparities. Of note, our study, which involved recruitment from the community rather than a school or clinic, had the lowest retention rate -possibly suggesting differences in the nature of the therapeutic relationship may matter.

In contrast to studies focusing on CWMT in adults, those in children tend to have higher completion rates, though still with significant variation across studies (6–26%). This is not surprising as Cogmed was initially developed for children, where the support of a teacher or parent is included to promote treatment compliance. Chacko et al.'s. (34) review examining the efficacy of CWMT for youth emphasizes that child users' training is monitored by both a parent/caregiver and a coach, highlighting that both roles are “essential particularly…where motivational issues and/or oppositional behavior may detract from compliance to CWMT.” In adult implementation of CWMT however, coaching is offered once a week via phone at best, likely contributing to observed differences in adherence and completion.

## Conclusion and future directions

The present perspective draws attention to possible concerns regarding the readiness of CMWT for usage in adults with ADHD. Review of the literature, combined with our own findings, revealed notable variation in the tolerability of CWMT for adults with ADHD. It is important to emphasize the need for conducting ecologically valid research before implementing interventions in the community. This is especially so when costs and burden of potential interventions are high-the CWMT fee-structures for patients in the community require payment in full (often between $1,000 and $2,500) to access the program, which is often not covered or reimbursed by insurance. Moreover, adult CWMT users are faced with unique scheduling challenges, such as work and family commitments, highlighting the need for treatments that are both efficacious and efficient. CWMT touts having been developed by “leading neuroscientists” using a top-down expert-driven method, but to our knowledge no empirical end-user validation research for Cogmed exists. Active end-user involvement in development can prevent deployment of intolerable training models. CWMT may still be a promising solution for current issues surrounding accessibility and efficacy of adult ADHD therapies, though it appears that significant examination and refinement will likely be needed for it to realize its potential.

## Ethics statement

This study was carried out in accordance with the recommendations of Institutional Review Board at the Nathan Kline Institute, with written informed consent from all subjects. All subjects gave written informed consent in accordance with the Declaration of Helsinki. The protocol was approved by the Institutional Review Board at the Nathan Kline Institute.

## Author contributions

EM: data acquisition, data organization, data analysis, drafting of manuscript. EH: data acquisition, data organization, drafting of manuscript. MK: data acquisition. LA, FC, and MPM: experiment design, drafting of manuscript.

### Conflict of interest statement

The authors declare that the research was conducted in the absence of any commercial or financial relationships that could be construed as a potential conflict of interest.
